# Anatomically Accurate 3D-Printed Thoracic Model With Artificial Skin for Chest Drainage Training

**DOI:** 10.7759/cureus.106964

**Published:** 2026-04-13

**Authors:** Rene D Mileva-Popova, Konstantinos Papadakis, Krasimir K Yanev, Andrey D Petrov, Todor G Bogdanov

**Affiliations:** 1 Department of Physiology and Pathophysiology, Medical University, Sofia, BGR; 2 Department of Dermatology and Venerology, Medical University, Sofia, BGR; 3 Department of Pharmacology and Toxicology - Clinical Pharmacology and Therapeutics Sector, Medical University, Sofia, BGR; 4 Medical Physics, Medical University, Sofia, BGR

**Keywords:** anatomical model, chest tube, low-cost simulator, medical education, procedural simulation, silicone-based artificial skin, simulation-based training, surgical skills training, thoracentesis, three-dimensional printing

## Abstract

Simulation-based training is an essential component of modern medical education, particularly for invasive procedures such as chest tube insertion and thoracentesis, where incorrect technique may lead to serious complications. The aim of this technical report is to describe the development and implementation of an anatomically accurate thoracic training model created using computed tomography (CT) data, three-dimensional (3D) printing, and silicone molding technologies. CT data from an anonymized institutional imaging database were segmented to create a three-dimensional model of the rib cage, which was fabricated using fused deposition modeling (FDM) with polylactic acid (PLA). The printed model included a right hemithorax with an integrated base structure allowing the placement of soft-tissue simulation layers and a fluid simulation system. Artificial skin was fabricated from two-component silicone with a thickness of 1-2 mm, and a 10 mm porous sponge layer was used to simulate subcutaneous tissue. The model also included a fluid-filled double-wall balloon maintained under pressure (1.1-1.2 atm) to simulate pleural fluid aspiration and flashback during needle puncture.

The model was designed for in-house manufacturing, allowing rapid production, low cost, and adaptability for different training scenarios. The manufacturing time for the 3D-printed thoracic structure was between 2 and 4 hours, while the artificial skin required up to 24 hours for curing. The total material cost per model ranged between 5 and 8 EUR. The model was reusable and allowed multiple chest tube insertions in different intercostal spaces.

The model was implemented in simulation-based training sessions involving 50 medical students from the second to fourth year of study. Students worked in small groups and performed the full procedure, including identification of anatomical landmarks, skin incision, blunt dissection, chest tube insertion, and surgical fixation. The educational value of the model was evaluated using a questionnaire based on a 5-point Likert scale. Student evaluation demonstrated a high level of satisfaction, with an overall mean score of 4.61 ± 0.50, indicating high perceived realism, usefulness, and educational value.

The presented model provides a low-cost, anatomically accurate, and reusable training platform that allows simulation of multiple thoracic procedures using a single model. The possibility for in-house manufacturing allows rapid prototyping, customization, and repeated use, making the model suitable for undergraduate medical education, procedural skills training, and simulation-based learning environments.

## Introduction

Chest tube insertion and thoracentesis are essential clinical procedures performed in emergency medicine, surgery, pulmonology, and critical care. These procedures are used to manage pneumothorax, hemothorax, pleural effusion, and other thoracic conditions, and improper technique may lead to serious complications, including organ injury, bleeding, and infection. Because of these risks, procedural training in a controlled and safe environment is essential before performing these interventions on patients [1,2].

Traditionally, procedural training has followed the apprenticeship model of “see one, do one, teach one.” Still, modern medical education increasingly emphasizes simulation-based training to improve procedural skills while minimizing risk to patients. Simulation-based education has been shown to improve technical skills, procedural confidence, and clinical performance, making it an important component of undergraduate and postgraduate medical training [3]. In particular, thoracic procedures such as chest tube insertion require the development of tactile skills, anatomical orientation, and procedural sequencing, which can be effectively taught using simulation models [4].

Commercially available training simulators exist; however, they are often expensive and not accessible to many medical schools, especially in low-resource settings. Additionally, many commercial models have limited anatomical realism or restricted possibilities for procedural variation. As a result, there has been increasing interest in the development of low-cost, high-fidelity training models using three-dimensional (3D) printing technologies [5, 6]. Three-dimensional printing allows the creation of anatomically accurate models based on computed tomography (CT) data, enabling the production of patient-specific anatomical replicas suitable for procedural training and medical education [7].

Recent studies have demonstrated the effectiveness of 3D-printed models in medical education and procedural training, showing improved student understanding, higher satisfaction, and improved psychomotor skill acquisition compared to traditional teaching methods alone [3]. Several authors have described 3D-printed thoracic models for chest tube insertion and pleural procedures, often combining rigid printed structures with flexible soft-tissue components to improve realism [4,8,9]. These models vary in complexity, cost, and manufacturing time, but most aim to provide a realistic and accessible alternative to cadaver-based or commercial simulation models.

Despite these developments, many existing models are either time-consuming to manufacture, relatively expensive, or designed to simulate only a single procedure. There remains a need for a flexible, low-cost, anatomically accurate training model that allows repeated practice of multiple thoracic procedures, including chest tube insertion, surgical fixation, and needle thoracentesis, within a single training platform. In-house production of simulation models allows rapid prototyping, customization, and scenario variability, making it a practical approach for medical universities and training centers seeking accessible simulation-based education tools.

This technical report aims to describe the development of an anatomically accurate thoracic training model created using a combination of 3D printing and silicone molding technologies. The model includes a 3D-printed rib cage derived from CT imaging data, an artificial silicone skin simulating human tissue properties, a subcutaneous layer, and a fluid simulation system that can reproduce pleural fluid flashback under pressure. The model was designed for in-house manufacturing to allow rapid production, low cost, and adaptability for different training scenarios. The model was implemented in simulation-based training sessions for medical students, and its educational value was evaluated using a student satisfaction questionnaire.

The novelty of the proposed model lies in the integration of multiple thoracic procedures within a single anatomically accurate platform, combined with ultra-low production cost and feasibility for rapid in-house manufacturing.

## Technical report

CT data processing and 3D model preparation

**Figure 1:**
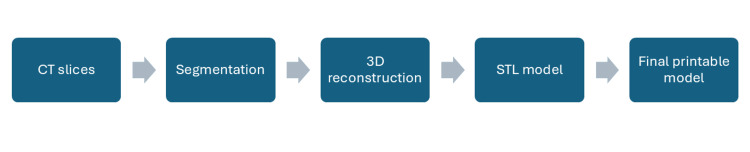
Workflow for CT data processing and 3D model generation. Image credit: Created using the Text-to-SmartArt function in Microsoft PowerPoint.

The anatomical model of the thoracic cage was developed using computed tomography (CT) data obtained from an anonymized institutional imaging database. The DICOM data were processed using medical image segmentation software to isolate the rib structures and generate a three-dimensional reconstruction of the thoracic anatomy. The segmentation workflow included threshold-based bone segmentation, manual refinement, and conversion of the segmented structures into a stereolithography (STL) file suitable for three-dimensional printing. The workflow for CT data processing and model generation is presented in Figure 1 (the graphical representation of the workflow was created using the Text-to-SmartArt function in Microsoft PowerPoint. The segmentation and model preparation methodology followed a previously described workflow for medical 3D model reconstruction [10].

To optimize the model for procedural training, only the right hemithorax was selected and processed. The rib cage model was digitally modified to include a supporting base structure beneath the ribs, forming a container that allows placement of additional simulation components, including subcutaneous material and a fluid simulation system.

3D printing of the thoracic cage

The thoracic model was fabricated using fused deposition modeling (FDM) technology with a Bambu Lab X1C 3D printer (Bambu Lab, Shenzhen, China). The model was printed using polylactic acid (PLA) filament with a 0.4 mm nozzle and a layer height of 0.2 mm. The printing time ranged between 2 and 4 hours, depending on the size of the printed thoracic segment. The average material cost for printing a single thoracic model ranged between 5 and 8 EUR, depending on the volume of the printed structure.

**Figure 2:**
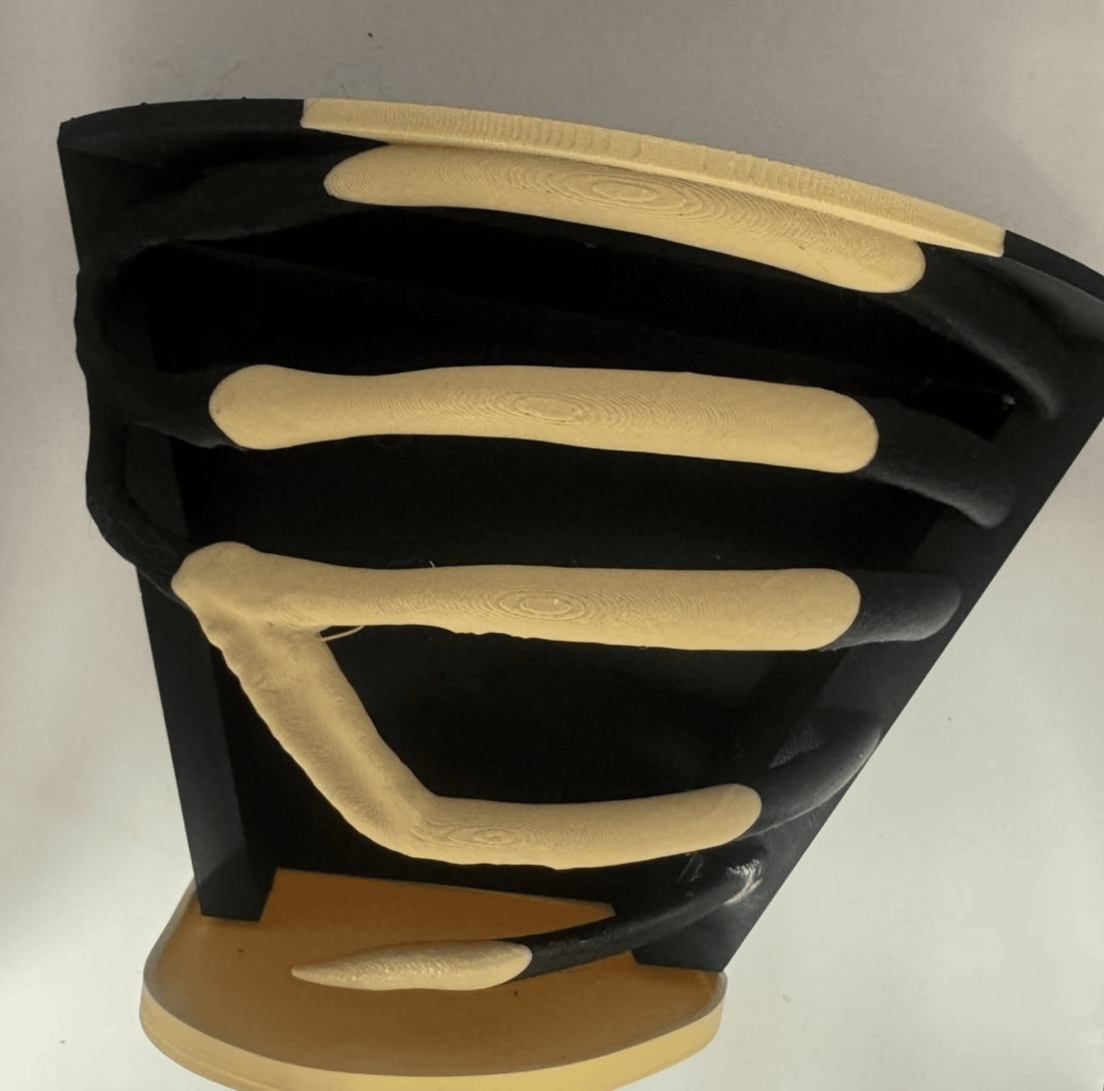
3D-printed thoracic model with integrated base platform. The model includes anatomically accurate rib structures derived from CT data and a supporting base that serves as a container for the soft tissue simulation layers and fluid system. Image credit: Todor Bogdanov.

The printed model included the ribs and an integrated base platform that served as a container for the soft tissue simulation layers and the fluid simulation system. The 3D-printed rib cage model is shown in Figure 2.

Artificial skin fabrication

**Figure 3:**
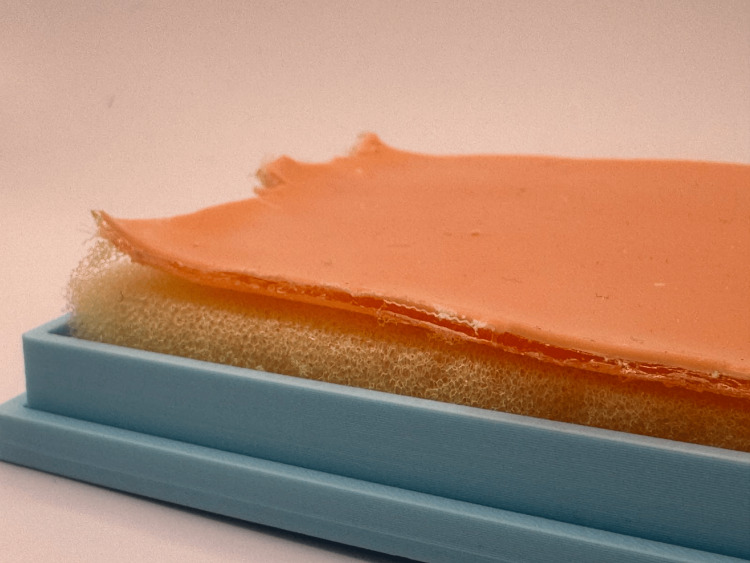
Fabricated silicone artificial skin used to simulate the mechanical properties of human skin. The material (two-component silicone) was molded to achieve a thickness of 1–2 mm, providing elasticity and resistance suitable for incision and suturing procedures. Image credit: Todor Bogdanov.

To simulate the mechanical properties of human skin, an artificial skin layer was fabricated using a two-component silicone material (Ecoflex 00-30, Smooth-On, Macungie, PA). The silicone was poured into a mold designed to produce a skin layer with a thickness between 1 and 2 mm. The silicone mixture was left to cure for up to 24 hours to achieve the desired mechanical properties and elasticity. The artificial skin fabrication process is illustrated in Figure 3. The artificial skin fabrication method and material properties were based on a previously developed artificial skin model designed for surgical training [11].

Model assembly and layered structure

The final training model consisted of multiple layers simulating the anatomical structure of the thoracic wall. A porous sponge material with a thickness of approximately 10 mm was placed between the artificial skin and the rib cage to simulate subcutaneous tissue and provide realistic resistance during incision and blunt dissection. The artificial skin layer was positioned above the sponge layer and fixed to the rib structure.

**Figure 4:**
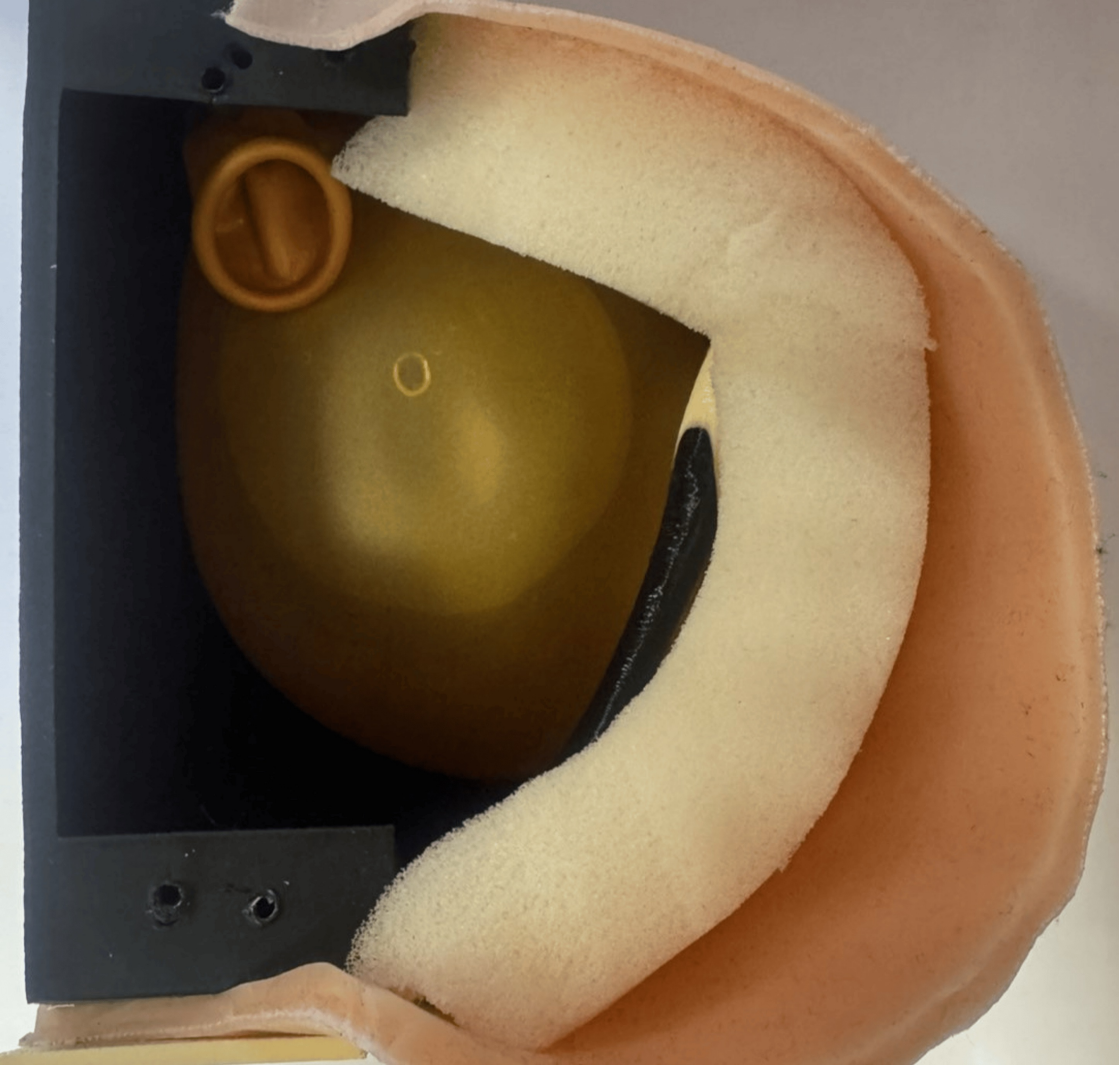
Layered structure of the thoracic training model showing the relationship between the artificial silicone skin, subcutaneous sponge layer, and underlying rib cage, designed to simulate realistic tactile resistance during procedural training. Image credit: Todor Bogdanov.

The base container beneath the ribs allowed the placement of additional simulation components, including a fluid-filled container used for procedural simulation. The layered structure of the model is shown schematically in Figure 4.

Fluid simulation system

**Figure 5:**
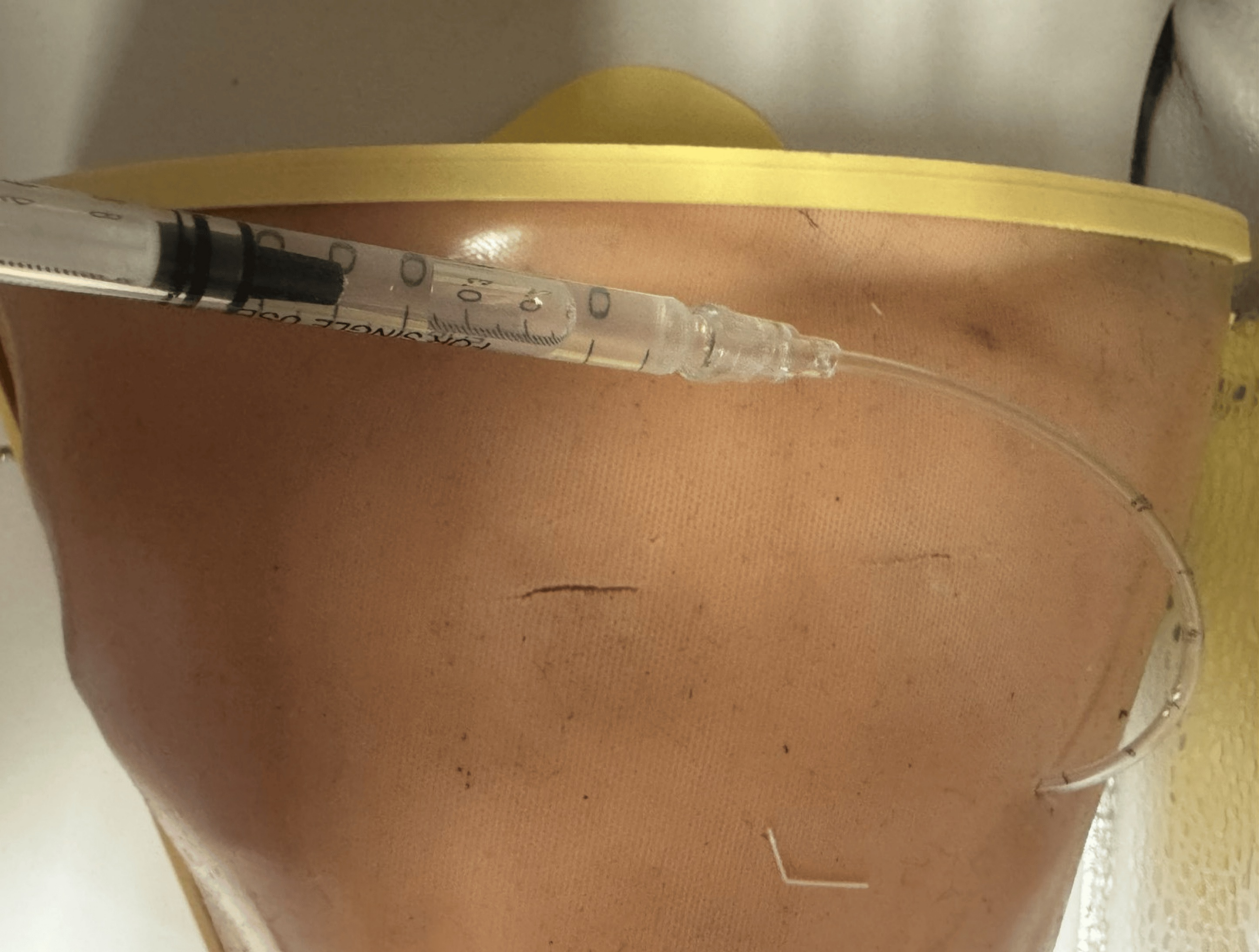
Pressurized fluid simulation system used to replicate pleural fluid aspiration during thoracentesis. A double-wall balloon filled with fluid is maintained under pressure (approximately 1.1-1.2 atm), producing a flashback effect upon needle puncture. Image credit: Krasimir Yanev.

The model was designed to simulate pleural fluid aspiration and needle thoracentesis. A double-wall balloon filled with fluid was placed inside the base container beneath the rib cage. The fluid inside the balloon was maintained under a pressure of approximately 1.1-1.2 atm. When punctured with a needle, the pressurized fluid produced a flashback effect, simulating pleural fluid aspiration during thoracentesis. The fluid simulation system is shown in Figure 5.

Training protocol

The model was used in simulation-based training sessions for medical students. A total of 50 medical students in their second to fourth year of study participated in the training. The students were divided into groups of five, with each group working with one thoracic model. Each training session lasted approximately 90 minutes and began with an instructor demonstration of the procedure and explanation of the relevant anatomical landmarks, including identification of the appropriate intercostal space along the midaxillary line.

**Figure 6:**
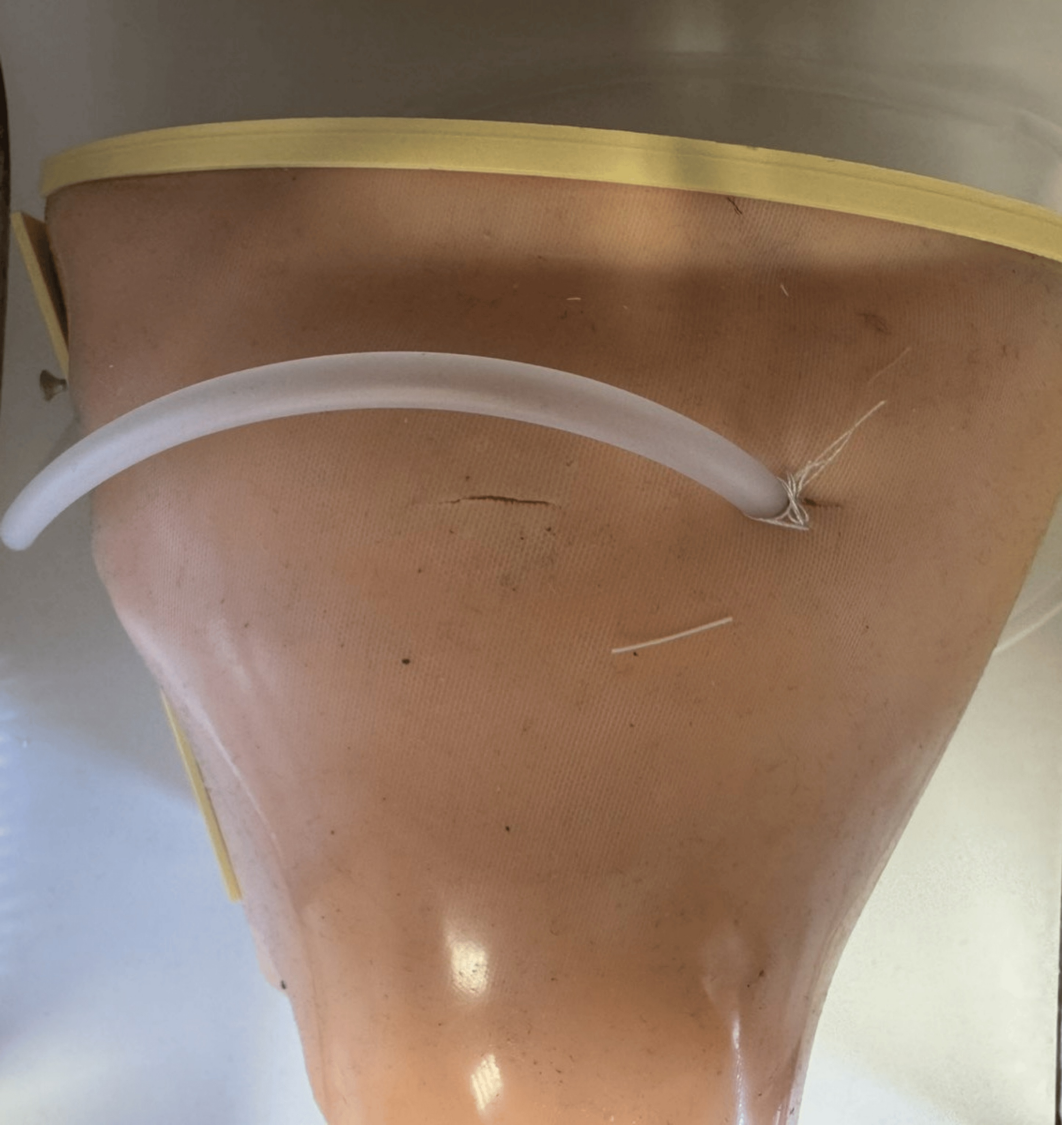
Chest tube insertion and fixation performed on the training model. The model allows identification of anatomical landmarks, incision, blunt dissection, tube placement, and fixation, simulating the complete procedural workflow. Image credit: Krasimir Yanev.

During the practical session, students performed the complete procedure, including skin incision with a scalpel, blunt dissection through the subcutaneous layer, insertion of a chest drainage tube, and surgical fixation of the tube with sutures. The model allowed placement of chest tubes in different intercostal spaces, enabling repeated practice. Each model could be used multiple times, typically allowing up to three chest tube placements in different intercostal spaces. The chest tube placement procedure performed on the model is shown in Figure 6.

Although the model was primarily used for chest tube insertion training, it was also designed to allow needle thoracentesis using the fluid simulation system.

Evaluation method

Following the training session, students were asked to complete an anonymous questionnaire evaluating the realism, usefulness, and educational value of the training model. The questionnaire consisted of 10 statements assessed on a 5-point Likert scale, where 1 indicated strong disagreement, and 5 indicated strong agreement. The items evaluated included anatomical realism, tactile feedback, usefulness for procedural training, identification of anatomical landmarks, confidence improvement, and overall satisfaction. All participating students completed the questionnaire, resulting in a response rate of 100%. The questionnaire results were analyzed using descriptive statistics, and mean values and standard deviations were calculated for each question. The results of the questionnaire are presented in Table 1. The evaluation of the model is based on subjective student feedback using a Likert-scale questionnaire and does not include objective performance-based metrics. Therefore, the results should be interpreted as preliminary indicators of perceived educational value rather than definitive evidence of training effectiveness. The questionnaire included items assessing anatomical realism, tactile feedback, usefulness for procedural training, confidence improvement, and overall satisfaction.

**Table 1:** Student questionnaire results (n = 50). SD, standard deviation

Question	Mean	SD
Q1. Anatomical realism	4.55	0.52
Q2. Tactile realism	4.48	0.58
Q3. Usefulness for chest tube training	4.67	0.47
Q4. Anatomical landmarks	4.6	0.49
Q5. Artificial skin realism	4.5	0.55
Q6. Confidence improvement	4.62	0.5
Q7. Repeated practice	4.7	0.46
Q8. Training usefulness	4.66	0.48
Q9. Should be included in the curriculum	4.68	0.47
Q10. Overall satisfaction	4.64	0.51

## Discussion

Model characteristics, manufacturing time, and cost

The developed thoracic training model combined a 3D-printed rib cage, artificial silicone skin, a subcutaneous layer, and a fluid-simulation system designed to replicate pleural fluid aspiration. The rib cage was printed using FDM with PLA filament, while the soft-tissue layers were fabricated from silicone and porous foam.

The manufacturing time for the 3D-printed thoracic structure ranged from 2 to 4 hours, depending on the size of the model. The artificial silicone skin required up to 24 hours for curing. The total material cost for producing one model ranged between 5 and 8 EUR, depending on the size of the thoracic segment and the amount of silicone used. The model could be reused multiple times, typically allowing up to three chest tube insertions in different intercostal spaces before the skin layer required replacement.

Compared to previously described thoracic simulators, the present model demonstrated significantly reduced manufacturing time and cost. Chen et al. reported a manufacturing time of approximately 118 hours and a cost of approximately USD 97 for a 3D-printed thoracostomy trainer [9], while Brannan et al. reported a production cost of approximately CAD 180 for a multifunctional thorax simulator [8]. In contrast, the model described in this study required only a few hours of printing time and had a material cost of less than 10 EUR, making it suitable for rapid in-house production and repeated use in student training. This rapid manufacturing capability allows institutions to produce multiple models in a short time and adapt model designs based on training requirements.

Low-cost simulation models are particularly important for medical schools and training centers with limited resources. Previous studies have emphasized that the cost of commercial simulators can be a major limitation for widespread implementation of simulation-based training, with commercial thoracentesis simulators costing between USD 2,500 and USD 4,000 [2]. The significantly lower cost of the model presented in this study allows repeated use and wider implementation in undergraduate training programs.

Training implementation and educational value

The model was used in simulation-based training sessions involving 50 medical students from the second to fourth year of study. Students worked in groups of five during 90-minute training sessions that included an instructor demonstration followed by hands-on procedural practice. Students performed all procedural steps, including identification of anatomical landmarks, skin incision, blunt dissection, chest tube insertion, and surgical fixation of the tube.

The layered structure of the model, consisting of silicone skin, a subcutaneous sponge layer, and a rigid rib cage, provided realistic tactile feedback during incision and dissection. The presence of anatomical rib structures allowed students to palpate and identify intercostal spaces, which is a critical step in chest tube placement. The model also allowed repeated practice by placing chest tubes in different intercostal spaces.

In addition to chest tube insertion, the model was designed to allow needle thoracentesis using a pressurized fluid system. The use of a fluid-filled balloon under pressure enabled the simulation of pleural fluid flashback during needle puncture, an important clinical indicator in thoracentesis. This feature enables the simulation of multiple thoracic procedures using a single model.

Simulation-based training has been shown to improve procedural skills, confidence, and clinical performance among medical students and residents [3]. The use of anatomically accurate 3D-printed models further improves spatial understanding, procedural orientation, and hands-on skill acquisition compared to traditional teaching methods alone [7]. Comparative studies between physical and virtual simulation models have shown that 3D-printed models are particularly effective for developing fine motor skills and procedural dexterity due to the realistic tactile interaction with physical structures. In contrast, virtual reality simulation is better suited to cognitive training and procedural planning [12]. These findings support the use of physical simulation models for procedural training, such as chest tube insertion, where tactile feedback and force control are critical.

Student evaluation

Student evaluation demonstrated a high level of satisfaction with the training model. The overall mean evaluation score was 4.61 ± 0.50 on a 5-point Likert scale, indicating high perceived realism, usefulness, and educational value of the model. The highest scores were associated with the capability for repeated practice and usefulness for chest tube training. In contrast, slightly lower scores were associated with tactile realism, as expected when synthetic materials are used instead of biological tissue.

These findings are consistent with previous studies evaluating 3D-printed simulation models, which have reported high student satisfaction and improved learning outcomes with simulation-based training [3]. Similar studies evaluating thoracic procedure simulators have also reported high satisfaction and perceived realism among trainees [4,8]. Although all evaluated domains received high scores, slightly lower ratings were observed for tactile realism, reflecting the inherent limitations of synthetic materials in fully replicating human tissue properties.

Advantages of the proposed model

The main advantage of the model described in this study is the integration of several features into a single training platform: anatomically accurate rib structures derived from CT data, artificial skin with realistic mechanical properties, a subcutaneous layer that allows blunt dissection, and a fluid simulation system that enables thoracentesis simulation. Many previously described models simulate only a single procedure, while the present model allows simulation of multiple thoracic procedures, including chest tube insertion, surgical fixation, and needle thoracentesis. High-fidelity 3D-printed thoracic models have also been used to experimentally simulate and test chest drainage systems under controlled conditions, demonstrating that 3D-printed models can reliably reproduce thoracic mechanics and procedural conditions [13]. This further supports the use of anatomically accurate 3D-printed thoracic models as effective tools for procedural training, device testing, and medical education.

Another important advantage is the ability to manufacture in-house. In-house production allows rapid prototyping, modification of model geometry, replacement of damaged components, and adaptation of the model for different training scenarios. This flexibility is not available with most commercial simulators. Furthermore, the low production cost allows the creation of multiple models, enabling training in small groups and increasing hands-on practice time for students.

Limitations

The present model has several limitations. Although the silicone skin and sponge layer provide realistic tactile feedback, they cannot fully replicate the mechanical properties of human tissue. Additionally, the model does not simulate bleeding or dynamic respiratory movement. The pressurized fluid system simulates fluid flashback but does not replicate all physiological conditions present during real thoracic procedures. Future improvements may include incorporating additional soft-tissue layers, ultrasound compatibility, or dynamic pressure control systems. The evaluation of the model is based on subjective student feedback obtained through a Likert-scale questionnaire and does not include objective performance-based metrics. The analysis is limited to descriptive statistics without inferential analysis or control groups, which restricts the ability to draw definitive conclusions regarding educational effectiveness. Therefore, the results should be interpreted as preliminary indicators of perceived educational value rather than definitive evidence of training effectiveness. Future studies incorporating objective assessment methods would be valuable to further validate the model.

Educational implications

Despite these limitations, the model provides a practical and accessible solution for simulation-based training in thoracic procedures. The combination of low cost, rapid production, anatomical accuracy, and procedural realism makes the model suitable for undergraduate medical education and skills training laboratories. The ability to produce the model in-house allows institutions to implement simulation-based training programs without the need for expensive commercial simulators.

## Conclusions

This technical report presents the development of an anatomically accurate thoracic training model, created using a combination of 3D printing and silicone molding. The model includes a CT-based 3D-printed rib cage, artificial silicone skin, a subcutaneous layer, and a pressurized fluid simulation system, allowing simulation of chest tube insertion, surgical fixation, and needle thoracentesis. The model demonstrated low production cost, rapid manufacturing time, and reusability, making it suitable for repeated use in undergraduate medical training. Student evaluations indicated high levels of satisfaction, perceived realism, and educational usefulness.

This technical report presents the development of an anatomically accurate thoracic training model combining 3D printing and silicone-based soft tissue simulation. The model demonstrates low production cost, rapid manufacturing time, reusability, and high levels of student satisfaction. The findings indicate that the model is a feasible and practical tool for simulation-based training and is well perceived by learners. However, the evaluation is based on subjective feedback, and therefore the results should be interpreted as indicators of perceived educational value rather than definitive evidence of training effectiveness. Future studies incorporating objective performance-based assessments are warranted to further evaluate the educational impact of the model.
